# EXPLORING THE EXPERIENCES OF PERCEIVED NEIGHBORHOOD SAFETY OF BLACK ADULTS: RESULTS FROM THE THINK PHRESH STUDY

**DOI:** 10.1093/geroni/igad104.3335

**Published:** 2023-12-21

**Authors:** Omar Staben, Alina Palimaru, Wendy Troxel, Tamara Dubowitz, Andrea Rosso

**Affiliations:** RAND Corporation, Pittsburgh, Pennsylvania, United States; RAND Corporation, Santa Monica, California, United States; RAND Corporation, Pittsburgh, Pennsylvania, United States; RAND Corporation, Pittsburgh, Pennsylvania, United States; University of Pittsburgh, Pittsburgh, Pennsylvania, United States

## Abstract

Racial residential segregation is a complex socio-economic phenomenon that affects many communities across the U.S. Such residential segregation is a result of historic policies in the United States including redlining and inequitable urban redevelopment. Black adults who live in segregated areas face challenges and experience psychosocial stressors, including poverty, crime, and feeling unsafe in one’s neighborhood which are risk factors for many negative health and cognitive outcomes. To date, few studies have explored perspectives on experiences of racial residential segregation, and specifically, neighborhood safety trajectories that may relate to healthy aging. This qualitative study used data from a sample of participants in adulthood and older adulthood (aged 35-89, Mage= 64 years) from two predominantly black, low-income neighborhoods that have had different histories with regard to disinvestment and systemic racist policies. We used 60 in-depth interviews to explore perceptions around neighborhood safety trajectories and their perceived impact on aging outcomes. The results highlight several dimensions of perceived changes in neighborhood safety over time and how these are perceived to relate to aging outcomes, e.g., psychological stress. The findings also show a few differences between the two neighborhoods concerning policing strategies, individual behaviors, and resource availability, all of which may contribute to perceptions of safety. Implications of this work highlight the importance of developing culturally relevant interventions that target upstream social determinants of healthy aging and health disparities.

